# Benefits and harms of prostate cancer screening – predictions of the ONCOTYROL prostate cancer outcome and policy model

**DOI:** 10.1186/s12889-017-4439-9

**Published:** 2017-06-26

**Authors:** Nikolai Mühlberger, Kristijan Boskovic, Murray D. Krahn, Karen E. Bremner, Willi Oberaigner, Helmut Klocker, Wolfgang Horninger, Gaby Sroczynski, Uwe Siebert

**Affiliations:** 1 Institute of Public Health, Medical Decision Making and Health Technology Assessment, Department of Public Health, Health Services Research and Health Technology Assessment, UMIT - University for Health Sciences, Medical Informatics and Technology, Eduard-Wallnoefer-Zentrum 1, A-6060 Hall i.T, Austria; 2 Division of Health Technology Assessment, ONCOTYROL - Center for Personalized Cancer Medicine, Innsbruck, Austria; 3 Toronto Health Economics and Technology Assessment (THETA) Collaborative, University of Toronto, Toronto, ON Canada; 4 Toronto General Research Institute, Toronto General Hospital, Toronto, ON Canada; 5 Cancer Registry of Tyrol, Tirol Kliniken GmbH, Innsbruck, Austria; 6 Department of Urology, Medical University of Innsbruck, Innsbruck, Austria; 7 Center for Health Decision Science, Department of Health Policy and Management, Harvard T.H. Chan School of Public Health, Boston, MA USA; 8 Institute for Technology Assessment and Department of Radiology, Massachusetts General Hospital, Harvard Medical School, Boston, MA USA

## Abstract

**Background:**

A recent recalibration of the ONCOTYROL Prostate Cancer Outcome and Policy (PCOP) Model, assuming that latent prostate cancer (PCa) detectable at autopsy might be detectable by screening as well, resulted in considerable worsening of the benefit-harm balance of screening. In this study, we used the recalibrated model to assess the effects of familial risk, quality of life (QoL) preferences, age, and active surveillance.

**Methods:**

Men with average and elevated familial PCa risk were simulated in separate models, differing in familial risk parameters. Familial risk was assumed to affect PCa onset and progression simultaneously in the base-case, and separately in scenario analyses. Evaluated screening strategies included one-time screening at different ages, and screening at different intervals and age ranges. Optimal screening strategies were identified depending on age and individual QoL preferences. Strategies were additionally evaluated with active surveillance by biennial re-biopsy delaying treatment of localized cancer until grade progression to Gleason score ≥ 7.

**Results:**

Screening men with average PCa risk reduced quality-adjusted life expectancy (QALE) even under favorable assumptions. Men with elevated familial risk, depending on age and disutilities, gained QALE. While for men with familial risk aged 55 and 60 years annual screening to age 69 was the optimal strategy over most disutility ranges, no screening was the preferred option for 65 year-old men with average and above disutilities.

Active surveillance greatly reduced overtreatment, but QALE gains by averted adverse events were opposed by losses due to delayed treatment and additional biopsies. The effect of active surveillance on the benefit-harm balance of screening differed between populations, as net losses and gains in QALE predicted for screening without active surveillance in men with average and familial PCa risk, respectively, were both reduced.

**Conclusions:**

Assumptions about PCa risk and screen-detectable prevalence significantly affect the benefit-harm balance of screening. Based on the assumptions of our model, PCa screening should focus on candidates with familial predisposition with consideration of individual QoL preferences and age. Active surveillance may require treatment initiation before Gleason score progression to 7. Alternative active surveillance strategies should be evaluated in further modeling studies.

## Background

Prostate cancer (PCa) is the most frequently diagnosed male malignancy and the third most frequent cause of male cancer death in the WHO European Region [1]. In Austria about 5000 of the approximately 4.1 million male inhabitants are newly diagnosed each year and about 1100 die from the disease [2].

To date, prostate-specific antigen (PSA) screening is the only method to detect early asymptomatic PCa, with the aim of reducing PCa mortality and metastatic disease. However, the benefit of screening is still controversial, as evidence for mortality reduction from trials is conflicting, and potential gains in life expectancy are opposed by losses in quality of life (QoL) due to overdiagnosis and overtreatment. Due to the uncertain benefit, medical organizations in Europe and the United States, including the European Association of Urology and the United States Preventive Services Task Force (USPSTF), do not recommend routine PSA-based screening [3, 4].

Observational data from the Austrian state of Tyrol, where free PSA screening was introduced for men aged 45–74 years in 1993, suggest a 30% screening-related decline of PCa mortality [5–7]. However, evidence from clinical trials is conflicting. While the European Randomized Study of Screening for Prostate Cancer (ERSPC) demonstrated a 29% reduction in PCa mortality at 11 year follow-up [8] and a 30% reduction in metastatic disease at 12 year follow-up [9], other trials including the Prostate, Lung, Colorectal, and Ovarian (PLCO) cancer screening trial [10] as well as a meta-analysis of trials [11] did not reveal significant reductions in disease-specific mortality.

The potential gains of screening are opposed by reduced QoL related to diagnostic biopsies, burdensome cancer treatment, including radical prostatectomy (RP), radiotherapy (RT), and androgen deprivation therapy (ADT), and the frequent long-term treatment-related adverse events, including erectile dysfunction (ED), urinary incontinence (UI) and bowel dysfunction (BD) [12–14]. In addition, RP has a small risk of peri-operative death [12, 13].

Finally, since a substantial fraction of PCas shows late onset and slow progression, overdiagnosis and overtreatment are common consequences of screening, especially when it is performed repeatedly or in men with relatively short remaining life expectancy due to age or life-shortening co-morbidity. Overdiagnosis and overtreatment are difficult to assess in empirical studies, because it would require a lifelong follow-up of men randomized to no screening and various screening options in a study without migration bias. Because such a study is virtually impossible to conduct, simulation models are used to estimate and counterbalance the QoL trade-offs due to overdiagnosis and overtreatment. However, out of the more than 25 PCa screening models [15–41], developed since the early 1990s, only five [21, 29, 37, 40, 41] account for both QoL and overdiagnosis with inconclusive results. While three of the five models [21, 29, 37] including the Erasmus MIcrosimulation SCreening ANalysis (MISCAN) model predict gains in quality-adjusted life expectancy (QALE) by screening up to age 70 and above in men with average PCa risk, two models [40, 41] including the ONCOTYROL Prostate Cancer Outcome and Policy (PCOP) model predict potential losses. Estimates from the Erasmus MISCAN model suggest that overdiagnosis among screen-detected cancers might be as high as 50% [37, 42, 43]. Estimates from the ONCOTYROL PCOP Model indicate even higher percentages [41].

The original PCOP Model included a natural history and detection module based on the structure and calibrated parameters of an early version of the Erasmus MISCAN model [42]. However, comparison with data from autopsy studies suggested an underestimation of latent cancer prevalence, which may cause an underestimation of overdiagnosis and thus bias the model in favor of screening. Therefore, the model was recalibrated to match data from autopsy studies as well. Recalibration to higher prevalence, assuming that latent PCa detectable at autopsy might be detectable by screening as well, resulted in a considerable increase in overdiagnosis and decline in screening sensitivity, which shifted the benefit-harm balance of screening from QALE gains to losses [41]. However, previous analyses with the PCOP Model primarily focused on the effect of prevalence assumptions on the benefit-harm balance of screening and did not represent a comprehensive benefit-harm analysis. In particular, previous analyses did not take into account that the benefit-harm balance of screening is influenced by PCa risk, individual QoL preferences, age, and specifics of the screening algorithm, including different ages and intervals and the option to combine screening with active surveillance. The latter is considered to be a measure to break the link between overdiagnosis and overtreatment [3, 44–46]. However, as empirical evidence on active surveillance is still weak, it is an ideal technology to be explored by modeling.

In this work, we perform a comprehensive benefit-harm analysis using the recalibrated PCOP Model to address the following research questions. (i) Are there any screening strategies which yield a potential net gain in QALE for men with average PCa risk? (ii) What is the benefit-harm balance of screening in men with elevated familial PCa risk? (iii) What are the optimal screening strategies for men with average and familial PCa risk? (iiii) How do individual QoL preferences regarding complications of screening and age affect the optimal screening decision? (iiii) How does active surveillance affect the benefit-harm balance of screening?

## Methods

### The model

The Oncotyrol PCOP Model is a decision-analytic state-transition micro-simulation model simulating the natural history of PCa and the consequences of screening and treatment on duration and quality of life. The model is programmed in the software TreeAge Pro 2015 (TreeAge Software Inc., Williamstown, MA, USA). Model building considered international best practice recommendation for modeling [47, 48].

The model follows men from birth to death in annual time cycles. During their lifetime, men may develop latent cancer, which over time can progress in stage and grade. Cancer can be detected when symptoms occur, or earlier by screening, which our model assumes to consist of PSA testing followed by ultrasound guided sextant biopsy when PSA level is ≥3 ng/mL.

Detected cancers can be treated, with treatment choice and effectiveness depending on cancer stage. Our model assumes homogeneous stage-specific treatment with RP, RT and ADT for localized, regional and distant cancer, respectively, which is largely consistent with Tyrolean treatment patterns reported for 1993–2005 [49]. Adverse events of treatment considered by our model are ED, UI, BD and peri-operative mortality of RP. Treatment of localized and regional PCa may be curative, whereas treatment of distant cancer is considered to be only palliative.

Without cure, cancer may progress and result in disease-specific death, unless the man dies earlier from another cause. Adherence with screening, treatment, and active surveillance are assumed to be 100% to achieve benefit-harm predictions of intended screening strategies unaffected by external behavioral factors.

**Fig. 1:**
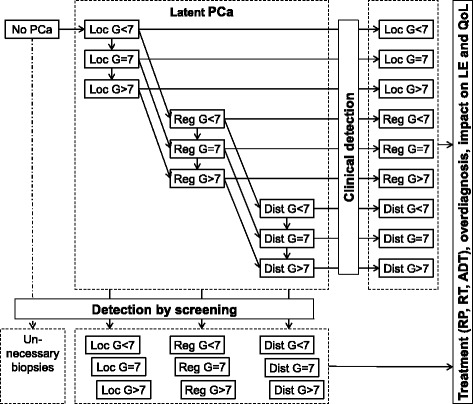
Structure of the ONCOTYROL PCOP Model. PCa: prostate cancer, Loc: localized cancer (T1/2, N0/X, M0/X), Reg: regional cancer (T3/4 or N+ and M0/X), Dist: distant cancer (any TN, M1), G: Gleason score, RP: radical prostatectomy, RT: radiotherapy, ADT: androgen deprivation therapy, LE: life expectancy, QoL: quality of life

Parameters of the natural history and detection component of the model are calibrated to data from autopsy studies, Dutch cancer registries, and the ERSPC. Structure and parameters of the model are presented in Fig. 1 and Table 1. Further details of the model, including its calibration and validation have been described earlier [41].

**Table 1:** Parameters of the Oncotyrol PCOP Model with annual time cycles

Parameters	Base-case values	Source
Natural history		
Prob. to exit the no cancer state (p / scale / shape)^a^	0.838 / 80.427/ 8.448	calibrated [41]
Prob. to exit local G < 7 cancer state (p / scale / shape)^a^	0.449 / 2.041 / 8.431	calibrated [41]
Prob. to exit local G = 7 cancer state (p / scale / shape)^a^	0.811 / 1.292 / 4.349	calibrated [41]
Prob. to exit local G > 7 cancer state (p / scale / shape)^a^	0.987 / 2.940 / 7.069	calibrated [41]
Prob. to exit regional G < 7 cancer state (p / scale / shape)^a^	0.450 / 6.050 / 4.129	calibrated [41]
Prob. to exit regional G = 7 cancer state (p / scale / shape)^a^	0.560 / 4.113 / 5.546	calibrated [41]
Prob. to exit regional G > 7 cancer state (p / scale / shape)^a^	0.823 / 2.024 / 2.791	calibrated [41]
Prob. to exit distant G < 7 cancer state (p / scale / shape)^a^	0.999 / 0.254 / 5.373	calibrated [41]
Prob. to exit distant G = 7 cancer state (p / scale / shape)^a^	0.945 / 0.806 / 4.564	calibrated [41]
Prob. to exit distant G > 7 cancer state (p / scale / shape)^a^	0.999 / 1.135 / 5.521	calibrated [41]
Familial risk factor on PCa onset and progression functions^a^	1.423	calibrated [41]
Prob. of local G < 7 cancer progress to regional	0.158	calibrated [41]
Prob. of local G = 7 cancer progress to regional	0.388	calibrated [41]
Prob. of regional G < 7 cancer progress to distant	0.005	calibrated [41]
Prob. of regional G = 7 cancer progress to distant	0.144	calibrated [41]
Prob. to die from PCa conditional on survival	SEER data	[53]
Age-specific prob. to die from other causes	Austrian life Table 2010/12	[54]
Cancer detection (clinically or by screening)		
Prob. of local G < 7 cancer to be clinically detected	0.006	calibrated [41]
Prob. of local G = 7 cancer to be clinically detected	0.110	calibrated [41]
Prob. of local G > 7 cancer to be clinically detected	0.604	calibrated [41]
Prob. of regional G < 7 cancer to be clinically detected	0.067	calibrated [41]
Prob. of regional G = 7 cancer to be clinically detected	0.108	calibrated [41]
Prob. of regional G > 7 cancer to be clinically detected	0.407	calibrated [41]
Prob. of distant G < 7 cancer to be clinically detected	0.233	calibrated [41]
Prob. of distant G = 7 cancer to be clinically detected	0.897	calibrated [41]
Prob. of distant G > 7 cancer to be clinically detected	1.000	Assumption
Prob. to participate in screening	1	Assumption
Prob. to detect local cancer by screening (Age < 70)	0.550	calibrated [41]
Prob. to detect local cancer by screening (Age 70+)	0.370	calibrated [41]
Prob. to detect regional/distant PCa by screening (Age < 70)	0.677	calibrated [41]
Prob. to detect regional/distant PCa by screening (Age 70+)	0.456	calibrated [41]
Spec. of PSA (to account for disutility by unnecessary biopsies)	0.85	[55]
Sens. of biopsy (to account for disutility by false neg. Biopsies)	0.90	[56, 57]
Spec. of biopsy	1	Assumption
Treatment (benefitial and harmful events)		
Probability of cure given local/regional cancer (G < 7)	0.51	[50]
Probability of cure given local/regional cancer (G = 7)	0.30	[50]
Probability of cure given local/regional cancer (G > 7)	0.11	[50]
Probability of cure given distant cancer (All G)	0	[58]
Risk to die from prostatectomy (30 Day mortality)	0.0015	[55]
Risk of erectile dysfunction attributable to prostatectomy	0.28	[13]
Risk of erectile dysfunction attributable to radiotherapy	0.15	[13]
Risk of urinary incontinence attributable to prostatectomy	0.22	[13]
Risk of urinary incontinence attributable to radiotherapy	0.031	[13]
Risk of bowel dysfunction attributable to prostatectomy	0	[13]
Risk of bowel dysfunction attributable to radiotherapy	0.028	[13]
Duration of treatment related dysfunctions	5 years	[59]
Utilities		
Utility without clinical distant PCa and treatment complication^b^	age-specific (1–0.78)	[60]
Utility of clinical distant cancer	0.6	[37]
Utility of erectile dysfunction by RP (PCI score 0–25)	0.89	[37, 59, 61]
Utility of erectile dysfunction by RT (PCI score > 25–50)	0.95	[37, 59, 61]
Utility of urinary incontinence by RP (PCI score > 50–75)	0.90	[37, 59, 61]
Utility of urinary incontinence by RT (PCI score > 75–100)	0.93	[37, 59, 61]
Utility of bowel dysfunction by RP (PCI score > 75–100)	0.93	[37, 59, 61]
Utility of bowel dysfunction by RT (PCI score > 75–100)	0.93	[37, 59, 61]
One-time relative utility for biopsy	0.994	calc. from [37]
One-time relative utility for RP	0.753	calc. from [37]
One-time relative utility for RT	0.772	calc. from [37]
One-time relative utility for terminal PCa	0.7	calc. from [37]

^a^Parameters for Eq. (1), ^b^ Age-specific utilities reported for the general male population

To address the research questions of this study, the previously published model was extended to include parameters for familial risk and active surveillance strategies with treatment delay and follow-up testing. In addition, we updated the cure rates based on the 29% PCa mortality reduction observed after 11 year follow-up of the ERSPC [8, 50], and replaced the constant additive one-time decrements for short-term disutility due to biopsy, RP and RT in our previous model with multiplicative one-time utility weights. The latter are more in favor of screening as the penalty for short-term disutility decreases with age-specific utility applied in our model.

### Familial risk

Results of a meta-analysis suggest that men with familial predisposition have at least a two-fold elevated PCa risk [51]. In our model familial risk is implemented as a weighting parameter on the time-dependent hazard function regulating the transitions from the no cancer state (i.e., PCa onset function) and the stage and grade specific cancer states (i.e., PCa progression functions) [41]. The hazard function and parameters of the function are shown in Eq. 1 and Table 1. The value for the familial risk parameter was calibrated in order to double the lifetime incidence of PCa of men in the pre-screening era from 9% to 18%. Because it is unclear how familial predisposition affects PCa onset and progression, our base-case analyses assumed a simultaneous effect on PCa onset and progression, while effects on PCa onset or progression alone were investigated in scenario analyses.

1 $$ h(t)\kern0.5em =\kern0.5em  fr\times \frac{p\left(\frac{a}{b}{\left(\frac{t}{b}\right)}^{a-1}{e}^{-{\left(\frac{t}{b}\right)}^a}\right)}{1- p\left(1-{e}^{-{\left(\frac{t}{b}\right)}^a}\right)} $$

, where.

fr = Familial risk factor.

p = Proportion of men at risk of exiting state (i.e. to latent cancer or next cancer state).

a = Weibull shape parameter.

b = Weibull scale parameter.

t = Time already spent in current health state.

### Active surveillance

Currently, there is no standard recommendation concerning eligibility, follow-up, and treatment initiation criteria for active surveillance. Our model assumes that men with screen-detected localized (T1/2, N0/X, M0/X) low grade (Gleason score < 7) cancer are eligible for active surveillance. Treatment is postponed until a grade progression to Gleason score ≥ 7 is detected by follow-up biopsy, which is performed in biennial intervals up to a maximum age of 74. Longer follow-up intervals were tested in exploratory analyses.

### Base-case analyses

Base-case analyses were performed from the perspective of 55 year old men confronted with the screening decision for the first time.

Men with average and elevated familial PCa risk were simulated in separate models, which were identical except for familial risk parameters. Models were analyzed using individual level simulation (microsimulation) with 10 million trials in order to track individual characteristics [48]. The time horizon for all analyses was lifetime (with an assumed maximum of 120 years).

Strategies evaluated by both models were no screening, one-time screening at age 55, 59, 64 and 69, and interval screening with 4, 2 and 1 year intervals at ages 55–59, 55–64, and 55–69. All interval screenings started at 55 years of age, as this was the age at which one-time screening performed best in terms of QALE in exploratory analyses. Interval screenings with higher starting ages were not considered in our base-case analyses as they can be expected to yield lower QALE. From age 55 we gradually extended the age window for interval screening in 5 years steps to age 69. We did not consider screening beyond age 69, as exploratory analyses revealed that potential benefits of screening decrease when the age window for screening is extended to age 74. To study the effect of active surveillance on the benefit-harm balance of screening all screening strategies were evaluated twice, once with immediate treatment and once with treatment postponed by active surveillance with biennial follow-up intervals.

Model outputs were chosen to cover the broad spectrum of screening-related benefits and harms, including frequencies of overdiagnosis, overtreatment and adverse events, PCa mortality, expected life days and quality-adjusted life days (QALDs), and numbers needed to screen to prevent one PCa death. QALDs gained versus no screening were our primary measure for the benefit-harm balance of screening.

In contrast to economic evaluations, our evaluation intends to provide personalized decision support for individual men, who are primarily interested in the real health effects of screening. Therefore, we did not apply any discounting.

### Scenario and sensitivity analyses

The effect of critical parameter assumptions on benefit-harm predictions was tested in scenario analyses applying more favorable parameter assumptions for screening. We considered scenarios with no peri-operative RP mortality, shorter duration of QoL impairment due to long-term complications of treatment (i.e., reduction from five years to one year), 50% lower one-time disutility weights for biopsy and curative treatment procedures, and non-age specific utilities for men without symptomatic metastatic cancer and treatment complications (i.e., assuming a utility of one instead of age-specific utilities from the general male population). In additional scenario analyses we restricted the effect of familial risk to PCa onset and PCa progression, respectively.

Sensitivity analysis was performed to study the effect of cure rate assumptions on the benefit-harm balance of the evaluated screening strategies in men with average PCa risk. In this analysis grade-specific cure-rates were increased simultaneously up to 80% via a common multiplicative factor. Sensitivity analysis was also used to investigate the impact of individual QoL preferences on expected QALDs of the screening strategies. Assuming that the preferences for the different long-term complications of treatment are linked within a person, we applied a common multiplier to the disutility weights (i.e., 1-Utility) for ED, UI and BD. This multiplier was varied between zero and two, where one indicates the average ED, UI and BD-related disutility weights applied in the base-case analysis, two reflects a disutility twice as high, and zero indicates no impairment from ED, UI and BD. Sensitivity analyses with varying QoL preferences were performed separately for screening candidates aged 55, 60 and 65 years to study the effect of age on the optimal screening decision as well.

### Overdiagnosis and overtreatment

Screen-detected cancers that would not progress to clinical stages during a man’s lifetime are considered to be clinically irrelevant and therefore represent overdiagnoses. Overdiagnosed men, who receive curative treatment (i.e., RP or RT) are considered to be overtreated [52].

Based on the assumption that only cancers detectable in the absence of screening by symptoms are clinically relevant, the difference in lifetime risks of cancer diagnosis with and without screening yields the lifetime risk of overdiagnosis. In addition, we present the fraction of overdiagnosis among screen-detected cancers, which is the risk of overdiagnosis divided by the risk of detection by screening. The same principle is applied to calculate overtreatment. It should be noted that even when diagnosis is followed by immediate treatment, figures for overdiagnosis and overtreatment deviate due to different reference figures for diagnosis and treatment in the no screening arms, which reflect the situation without overdiagnosis and overtreatment.

## Results

### Base-case analyses

Model predictions for men with average and elevated familial PCa risk are contrasted in Table 2.

**Table 2:** Base-case analyses - model predictions for men with average and elevated familial PCa risk

	No screening	One-time screening at age				Interval screening at age 55–59, with interval			Interval screening at age 55–64, with interval			Interval screening at age 55–69, with interval		
		55y	59y	64y	69y	4y (2 x)	2y (3 x)	1y (5 x)	4y (3 x)	2y (5 x)	1y (10 x)	4y (4 x)	2y (8 x)	1y (15 x)
Predictions for men with average PCa risk														
Lifetime risk of PCa diagnosis (%)	9.00	10.18	11.05	12.76	15.19	11.57	11.96	12.30	13.54	14.18	15.39	16.32	19.27	20.13
Lifetime risk of PCa diagnosis by screening (%)	-	1.46	2.53	4.55	7.37	3.24	3.76	4.22	5.78	6.67	8.32	9.35	13.33	14.56
Lifetime risk of overdiagnosis (%)	-	1.18	2.05	3.76	6.19	2.57	2.96	3.30	4.54	5.18	6.39	7.32	10.27	11.13
Overdiagnosis in screen-detected PCa (%)	-	80.82	81.03	82.64	83.99	79.32	78.72	78.20	78.55	77.66	76.80	78.29	77.04	76.44
Lifetime prob. of curative local treatment (%)	4.86	5.87	6.59	7.96	9.85	7.13	7.58	8.01	8.92	9.76	11.28	11.42	14.70	16.22
Lifetime prob. of curative regional treatment (%)	1.62	1.87	2.06	2.44	3.00	2.10	2.07	2.01	2.41	2.27	2.06	2.86	2.74	2.15
Lifetime prob. of curative loco/regional treatment (%)	6.48	7.74	8.65	10.40	12.85	9.23	9.65	10.02	11.33	12.03	13.34	14.28	17.44	18.37
Lifetime risk of curative overtreatment (%)	-	1.26	2.17	3.92	6.37	2.75	3.17	3.54	4.85	5.55	6.86	7.80	10.96	11.89
Curative overtreatment in screen-detected PCa (%)	-	86.30	85.77	86.15	86.43	84.88	84.31	83.89	83.91	83.21	82.45	83.42	82.22	81.66
Lifetime risk of dying of PCa (%)	1.72	1.69	1.67	1.64	1.63	1.64	1.62	1.60	1.58	1.54	1.47	1.51	1.39	1.30
Lifetime gained v. no screening (days)	-	0.9	1.1	2.2	1.9	1.9	2.2	2.4	3.3	4.3	7.2	5.8	8.7	12.3
**QALDs gained v. no screening**	**-**	**−1.3**	**−2.3**	**−3.9**	**−7.2**	**−2.8**	**−3.5**	**−4.4**	**−4.9**	**−5.8**	**−6.7**	**−7.3**	**−10.6**	**−11.2**
RP-related deaths per 10,000 men (*n*)	0.70	0.87	0.99	1.20	1.46	1.09	1.15	1.18	1.34	1.46	1.65	1.66	2.23	2.39
RP- and RT-related AEs per man (*n*)	0.029	0.034	0.038	0.046	0.057	0.041	0.044	0.046	0.051	0.056	0.063	0.065	0.083	0.090
PSA screening tests per man (*n*)	-	0.93	0.89	0.84	0.75	1.81	2.69	4.45	2.63	4.34	8.51	3.37	6.50	12.02
False-positive PSA tests per man (*n*)	-	0.13	0.13	0.11	0.09	0.26	0.39	0.66	0.38	0.63	1.25	0.48	0.94	1.76
PSA tests needed to avoid 1 death (*n*)	-	2994	1721	1135	848	2369	2774	3893	1964	2460	3382	1660	1964	2854
Men needed to be screened to avoid one death (*n*)	-	2994	1721	1135	848	1214	954	809	692	525	368	455	280	220
Predictions for men with elevated familial PCa risk														
Lifetime risk of PCa diagnosis (%)	18.00	19.28	20.23	22.08	24.64	20.79	21.22	21.58	22.93	23.62	24.94	25.92	29.07	30.03
Lifetime risk of PCa diagnosis by screening (%)	-	1.97	3.36	5.92	9.28	4.37	5.07	5.70	7.79	9.05	11.34	12.53	17.93	19.67
Lifetime risk of overdiagnosis (%)	-	1.28	2.23	4.08	6.64	2.79	3.22	3.58	4.93	5.62	6.94	7.92	11.07	12.03
Overdiagnosis in screen-detected PCa (%)	-	64.97	66.37	68.92	71.55	63.84	63.51	62.81	63.29	62.10	61.20	63.21	61.74	61.16
Lifetime prob. of curative local treatment (%)	8.61	9.73	10.53	11.98	13.88	11.18	11.75	12.31	13.23	14.37	16.39	16.04	20.15	22.37
Lifetime prob. of curative regional treatment (%)	3.69	4.07	4.35	4.92	5.73	4.40	4.34	4.22	4.88	4.60	4.16	5.55	5.22	4.13
Lifetime prob. of curative loco/regional treatment (%)	12.29	13.80	14.88	16.90	19.61	15.58	16.09	16.53	18.11	18.97	20.55	21.59	25.37	26.50
Lifetime risk of curative overtreatment (%)	-	1.51	2.59	4.61	7.32	3.29	3.80	4.24	5.82	6.68	8.26	9.30	13.08	14.21
Curative overtreatment in screen-detected PCa (%)	-	76.65	77.08	77.87	78.88	75.29	74.95	74.39	74.71	73.81	72.84	74.22	72.95	72.24
Lifetime risk of dying of PCa (%)	4.35	4.26	4.21	4.15	4.12	4.14	4.09	4.03	3.99	3.87	3.66	3.80	3.46	3.21
Lifetime gained v. no screening (days)	-	4.7	6.2	7.7	6.6	9.7	12.0	14.4	15.2	20.1	28.6	21.3	32.5	42.7
**QALDs gained v. no screening**	**-**	**1.8**	**1.7**	**0.3**	**−3.9**	**3.6**	**4.6**	**5.5**	**4.7**	**7.1**	**10.6**	**5.1**	**8.2**	**13.0**
RP-related deaths per 10,000 men (*n*)	1.32	1.49	1.64	1.79	2.12	1.73	1.82	1.84	2.01	2.18	2.38	2.39	3.07	3.28
RP- and RT-related AEs per man (*n*)	0.052	0.059	0.064	0.073	0.084	0.068	0.071	0.073	0.080	0.085	0.095	0.096	0.118	0.128
PSA screening tests per man (*n*)	-	0.92	0.89	0.82	0.73	1.79	2.67	4.40	2.60	4.28	8.34	3.32	6.34	11.66
False-positive PSA tests per man (*n*)	-	0.13	0.12	0.11	0.09	0.26	0.39	0.65	0.37	0.62	1.22	0.46	0.90	1.70
PSA tests needed to avoid 1 death (*n*)	-	1059	630	415	312	859	1021	1368	714	888	1205	605	712	1023
Men needed to be screened to avoid one death (*n*)	-	1059	630	415	312	442	353	287	254	192	133	168	104	81

Results are based on individual level simulation (microsimulation) with 10 million men. Time horizon = 120 years, Compliance = 100%. *PCa* prostate cancer, *QALD* quality-adjusted life day, *RP* radical prostatectomy, *RT* radiotherapy, *AE* adverse event

QALDs were primary benefit-harm outcome was indicated in bold

Predictions for men with average PCa risk shown in the upper section of the table indicate that the lifetime risk of PCa diagnosis increases with age and screening frequency, from 9% without screening to 20% with annual screening up to age 69. The lifetime risk of overdiagnosis increases in parallel up to 11%. Overdiagnosis expressed as a percentage of screen-detected cancers has a less consistent relationship to age and screening frequency with a minimum of 76% with annual screening up to age 69 and a maximum of 84% with one-time screening at age 69. Similar trends are predicted for curative loco/regional overtreatment.

All screening strategies are predicted to reduce PCa-specific mortality and increase lifetime. Mortality reductions by screening range from 2% with one-time screening at age 55 to 24% with annual screening up to age 69. Lifetime gains per man range from 0.9 days with one-time screening at age 55 to 12.3 days with annual screening up to age 69. One-time screening performs best at age 64, providing 2.2 extra days.

When QoL is taken into account, the benefit-harm balance of screening becomes negative. Losses in QALDs increase with screening age and frequency ranging from 1.3 QALD with one-time screening at age 55 to 11.3 QALDs with annual screening up to age 69.

The number of treatment-related adverse events increases with screening age and frequency as well. RP-related mortality increases from 0.7 deaths per 10,000 men without screening to 2.4/10,000 men with annual screening. RP- and RT-related adverse events, including ED, UI and BD, triple from 0.03 to 0.09 per man.

The number of PSA tests per man, including false positive tests that trigger unnecessary biopsies, decreases as the screening interval and age at screening increase. With one-time screening at age 69 the lifetime risk of a false positive PSA test is 9%, whereas with annual screening up to age 69 men would have more than one false positive test result in their lifetime. The number of PSA tests needed to prevent one PCa death was lowest with one-time screening at age 69 (848). The number of men needed to be screened was lowest with annual interval screening up to age 69 (220).

Predictions for men with elevated familial PCa risk are displayed in the lower section of Table 2. In contrast to men with average PCa risk, the model for men with familial predisposition shows a benefit-harm balance in favor of screening. All screening strategies, except one-time screening at age 69, are predicted to increase QALE. QALE gains increase with increasing screening frequency and length of the screening period to a maximum of 13 QALDs with annual screening to age 69. Apart from QALE gains, the model for men with elevated PCa risk predicts considerably higher lifetime gains, and fewer PSA tests or men needed to be screened to avoid one PCa death. Numbers of RP-related deaths and other RP- and RT-related adverse events are consistently higher compared to estimates for men with average PCa risk. In contrast, false positive PSA tests are less frequent. Lifetime risks of overdiagnosis and overtreatment are quite similar to those for men with average risk. This is a consequence of applying the familial risk parameter to both PCa onset and progression, which have been shown to influence the risk of overdiagnosis in opposite directions [41]. Different from lifetime risk estimates, percentages of overdiagnosis and overtreatment in screen-detected cancers are consistently lower in the familial risk model.

### Scenario and sensitivity analyses

Results of the scenario analyses evaluating the effect of critical model assumptions on QALDs gained versus no screening are presented in the Appendix.

Scenarios for screening in men with average PCa risk, which apply more favorable screening assumptions, still predict a negative benefit-harm balance, but with lower losses in QALE. Scenarios for screening in men with familial predisposition, applying more favorable screening assumptions, consistently yield higher gains in QALE except for one-time screening at age 69.

Scenario analyses investigating the effect of familial risk assumptions yield contrary results. When familial risk increases only PCa onset, the benefit-harm balance for men with familial predisposition becomes negative, whereas when only PCa progression is increased, the net benefit of screening considerably exceeds our base-case prediction.

**Fig. 2:**
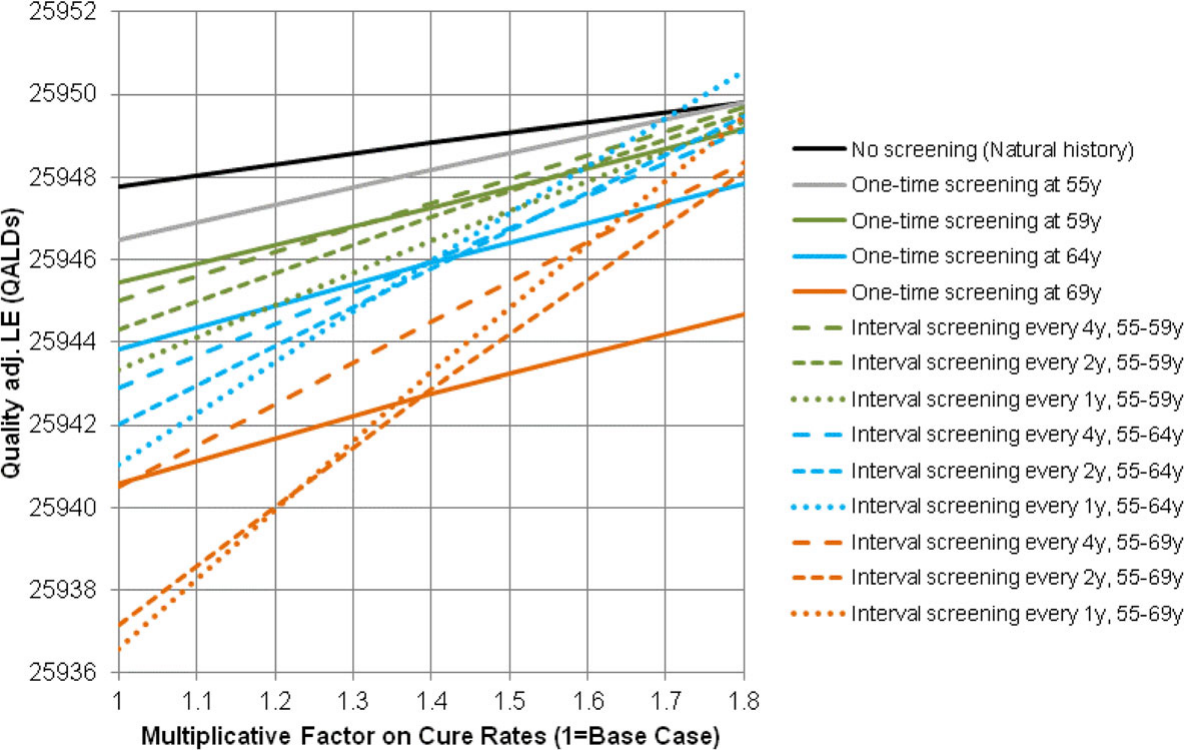
Sensitivity analyses on grade-specific cure rates in men with average PCa risk. Results are based on individual level simulation (microsimulation) with 10 million trials. Time horizon = 120 years, Compliance = 100%. PCa: prostate cancer, Quality adj. LE: Quality-adjusted life expectancy, QALD: quality-adjusted life day

Figure 2 presents the sensitivity analysis on grade-specific cure rates in men with average cancer risk. As cure rates increase the difference in QALE between no screening and screening decreases. However, cure rates have to be increased by more than 70% before screening becomes more beneficial.

**Fig. 3:**
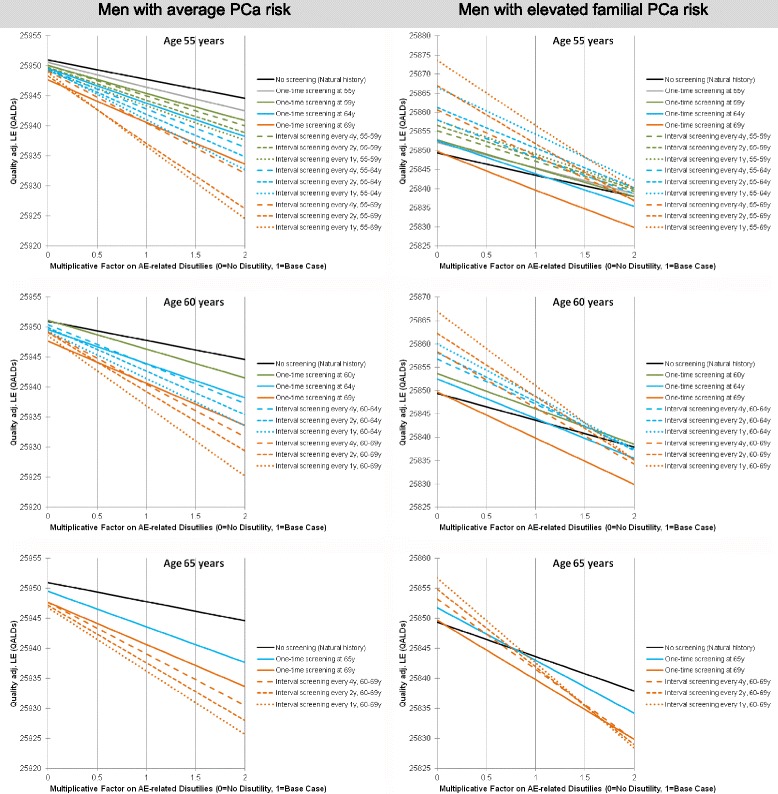
Sensitivity analyses on quality of life preferences and age. Results are based on individual level simulation (microsimulation) with 10 million trials. Time horizon = 120 years, Compliance = 100%. PCa: prostate cancer, Quality adj. LE: Quality-adjusted life expectancy, QALD: quality-adjusted life day

Sensitivity analyses investigating the effect of QoL preferences and age are summarized in Fig. 3, with 55, 60 and 65 year old men with average PCa risk on the left, and men with elevated familial risk on the right. Each colored line in the graphs indicates the prediction for one of our compared screening options. No screening is indicated by the black line.

No screening is the preferred option for screening candidates with average PCa risk, irrespective of age and individual utility weighting of potential long-term adverse events of curative PCa treatment (i.e. ED, UI, BD).

The optimal screening strategy for candidates with elevated familial risk varies with QoL preference and age. For 55 and 60 year old men annual screening to age 69 is the preferred option over most of the investigated preference range, including average base-case preference indicated by a value of one on the x-axis. A change in the optimal screening strategy is observed only in the men with 50% higher disutility. In 65 year old candidates no screening is the optimal strategy with base-case or higher disutility, and annual screening to 69 with lower disutility.

### Active surveillance

Table 3 presents model predictions for screening with biennial active surveillance follow-up. Results for men with average and elevated familial PCa risk are shown in the upper and lower sections of the table, respectively. Outcomes, which are not affected by active surveillance and therefore identical to base-case results, are excluded from the table.

**Table 3:** Screening with biennial active surveillance follow-up - model predictions for men with average and elevated familial PCa risk

	No screening	One-time screening at age				Interval screening at age 55–59, with interval			Interval screening at age 55–64, with interval			Interval screening at age 55–69, with interval		
		55y	59y	64y	69y	4y (2 x)	2y (3 x)	1y (5 x)	4y (3 x)	2y (5 x)	1y (10 x)	4y (4 x)	2y (8 x)	1y (15 x)
Predictions for men with average PCa risk														
Follow-up biopsies per man under active surveillance (*n*)	-	6.7	5.3	3.9	1.8	5.7	5.6	5.5	4.8	4.7	4.4	3.9	3.4	3.4
Lifetime prob. of curative local treatment (%)	4.86	5.17	5.39	5.81	6.37	5.55	5.66	5.85	6.09	6.27	6.90	6.84	7.65	8.45
Lifetime prob. of curative regional treatment (%)	1.62	1.88	2.08	2.46	2.94	2.12	2.11	2.08	2.45	2.34	2.23	2.89	2.76	2.34
Lifetime prob. of curative loco/regional treatment (%)	6.48	7.05	7.46	8.27	9.32	7.67	7.77	7.93	8.54	8.61	9.12	9.73	10.40	10.79
Lifetime risk of curative overtreatment (%)	-	0.57	0.98	1.79	2.84	1.19	1.29	1.45	2.06	2.13	2.64	3.25	3.92	4.31
Curative overtreatment in screen-detected PCa (%)	-	39.04	38.74	39.34	38.53	36.73	34.31	34.36	35.64	31.93	31.73	34.76	29.41	29.60
Lifetime risk of dying of PCa (%)	1.72	1.69	1.68	1.66	1.65	1.66	1.65	1.64	1.62	1.61	1.56	1.57	1.52	1.47
Lifetime gained v. no screening (days)	-	0.7	0.7	1.9	1.9	1.2	1.0	1.0	1.9	1.9	3.9	3.9	5.1	7.1
**QALDs gained v. no screening**	**-**	**−0.5**	**−0.9**	**−1.0**	**−2.1**	**−1.2**	**−1.9**	**−2.7**	**−2.0**	**−2.9**	**−3.6**	**−2.3**	**−3.7**	**−4.9**
RP-related deaths per 10,000 men (*n*)	0.70	0.76	0.81	0.86	0.94	0.83	0.83	0.87	0.90	0.91	1.04	1.02	1.15	1.26
RP- and RT-related AEs per man (*n*)	0.029	0.031	0.032	0.035	0.039	0.033	0.034	0.035	0.037	0.038	0.041	0.042	0.047	0.051
PSA tests needed to avoid 1 death (*n*)	-	4042	2251	1511	1145	3245	4098	5860	2742	3895	5482	2371	3278	4807
Men needed to be screened to avoid one death (*n*)	-	4042	2251	1511	1145	1663	1408	1218	966	831	596	651	467	370
Predictions for men with elevated familial PCa risk														
Follow-up biopsies per man under active surveillance (*n*)	-	5.9	4.8	3.6	1.7	5.1	4.9	4.7	4.3	4.1	3.8	3.5	3.1	2.9
Lifetime prob. of curative local treatment (%)	8.61	9.00	9.29	9.81	10.47	9.52	9.69	9.99	10.27	10.59	11.57	11.28	12.56	13.87
Lifetime prob. of curative regional treatment (%)	3.69	4.10	4.40	4.98	5.64	4.48	4.46	4.40	5.02	4.84	4.61	5.71	5.44	4.70
Lifetime prob. of curative loco/regional treatment (%)	12.29	13.10	13.69	14.79	16.11	14.01	14.16	14.39	15.29	15.43	16.19	16.99	18.00	18.57
Lifetime risk of curative overtreatment (%)	-	0.81	1.40	2.50	3.82	1.72	1.87	2.10	3.00	3.14	3.90	4.70	5.71	6.28
Curative overtreatment in screen-detected PCa (%)	-	41.12	41.67	42.23	41.16	39.36	36.88	36.84	38.51	34.70	34.39	37.51	31.85	31.93
Lifetime risk of dying of PCa (%)	4.35	4.29	4.25	4.20	4.18	4.20	4.18	4.14	4.09	4.05	3.93	3.97	3.82	3.68
Lifetime gained v. no screening (days)	-	3.5	4.7	6.2	5.4	7.1	8.0	9.7	10.8	12.8	18.0	15.3	20.6	26.3
**QALDs gained v. no screening**	**-**	**1.8**	**2.2**	**2.0**	**0.0**	**3.5**	**3.7**	**4.2**	**4.8**	**5.6**	**7.3**	**6.3**	**8.0**	**9.5**
RP-related deaths per 10,000 men (*n*)	1.32	1.39	1.44	1.49	1.59	1.47	1.48	1.53	1.57	1.60	1.74	1.72	1.90	2.07
RP- and RT-related AEs per man (*n*)	0.052	0.055	0.058	0.062	0.067	0.059	0.060	0.062	0.065	0.066	0.072	0.072	0.079	0.086
PSA tests needed to avoid 1 death (*n*)	-	1429	843	560	429	1196	1535	2055	1015	1417	1962	873	1192	1747
Men needed to be screened to avoid one death (*n*)	-	1429	843	560	429	615	531	431	360	306	217	243	173	138

Results are based on individual level simulation (microsimulation) with 10 million trials. Time horizon = 120 years, Compliance = 100%, Active surveillance interval = 2 years. *PCa* prostate cancer, *QALD* quality-adjusted life day, *RP* radical prostatectomy, *RT* radiotherapy, *AE* adverse event

QALDs were primary benefit-harm outcome was indicated in bold

Comparison with model predictions for screening without active surveillance (Table 2) indicates that, depending on the screening strategy, active surveillance reduces overtreatment by 54–64% and 46–56% in men with average and familial PCa risk, respectively. Associated reductions in RP-related deaths range from 23 to 49% and 7–38%, and reductions in RP- and RT-related long-term adverse events from 10 to 44% and 6–33% in men with average and familial risk, respectively.

The benefits of active surveillance are opposed by harms due to follow-up biopsies and delayed treatment. The number of active surveillance follow-up biopsies predicted per man with average and elevated familial PCa risk under active surveillance ranges from 1.8 to 6.7 and 1.7 to 5.9, respectively. The probability of curative loco/regional treatment is reduced by 9–41% and 5–30%, respectively, which is also illustrated by a shift from treating localized cancer to treating regional cancer. Consequently, screening with active surveillance consistently shows lower gains in lifetime and higher numbers needed to screen in both investigated screening populations.

However, the effect of active surveillance on QALE differs between populations. While QALE losses predicted for men with average PCa risk in the base-case analysis are consistently less pronounced with active surveillance, QALE gains predicted for men with familial risk tend to be lower when interval screening is combined with active surveillance.

## Discussion

In this work we used the decision-analytic ONCOTYROL PCOP Model to assess the benefit-harm balance of PCa screening in men with average and elevated familial risk, and studied the effects of individual QoL preferences, age, and active surveillance on the benefit-harm balance.

### Screening in men with average PCa risk

Our simulations suggest that screening in men with average PCa risk yields potential gains in life expectancy, but potential losses in QALE. Losses are predicted for all evaluated screening algorithms, including one-time screening at different ages and interval screenings with different screening intervals and age ranges, and in all scenario analyses applying more favorable screening assumptions. In addition, sensitivity analyses indicate that no screening remains the preferred option for screening candidates with average PCa risk, irrespective of their age and individual preference weighting of potential long-term adverse events of curative PCa treatment.

The QALE losses predicted by our model are in line with recent results from a Canadian model [40], but contradictory to the Erasmus MISCAN model, which indicated considerable gains by screening [37, 50]. The reasons for these contradictory results are not completely understood, in particular because the ONCOTYROL PCOP Model adopted structural elements of the natural history component and crucial assumptions, including cure rates and short-term disutility assumptions from the MISCAN model. Currently, the most likely explanation for the contradictory results are different assumptions about latent PCa prevalence, which can strongly affect overdiagnosis and thus the benefit-harm balance of screening, as shown by previous analyses [41].

### Screening in men with elevated familial PCa risk

For men with elevated familial PCa risk our model predictions are clearly in favor of screening. All screening strategies, except one-time screening at age 69, are predicted to increase QALE, and gains in unadjusted lifetime are consistently higher than in men with average PCa risk.

Base-case results suggest that QALE gains increase with screening frequency and length of the screening period up to a certain age, which is a mechanism shown by the MISCAN model as well [50]. However, sensitivity analyses indicate that the optimal screening decision also depends on individual QoL preferences and age. Apart from that, it should be noted that our analyses are performed for screening candidates with normal life expectancy. As the risk of overdiagnosis increases with declining life expectancy our results should not be applied to men with life-shortening co-morbidity.

Considering that one-time screening at age 69 results in loss of QALE, it seems odd that extending the upper age limit of interval screening from 64 to 69 years yields additional QALE. However, this indicates that with interval screening starting at age 55, men in the age range of 65 to 69 years are preselected by previous screenings, which may already have harvested most cases of overdiagnosis.

Prostate cancer screening in men with familial predisposition was previously simulated in an Australian model by Howard et al. [32]. Similar to our model, the Australian model indicated that the numbers of cancer diagnoses, averted deaths and screening-related harms are higher when screening is performed in a high risk population. Unlike in our model however, benefits and harms were not balanced against each other using a common metric like QALE, which is required to assess the net benefit of screening.

### Active surveillance

Active surveillance is considered to be a measure to break the link between overdiagnosis and overtreatment [44–46], but empirical evidence concerning its consequences is still weak. We simulated active surveillance with biennial follow-up biopsies in men with screen-detected localized, low grade cancer in whom treatment is postponed until detection of grade progression to Gleason score ≥ 7.

Predictions of our model indicate that active surveillance strongly reduces overtreatment. However, the effect of active surveillance on the benefit-harm balance of screening differs between the investigated screening populations. While predicted net QALE losses by screening in men with average PCa risk are consistently lower with active surveillance, highest net QALE gains for men with familial PCa risk are predicted with interval screening without active surveillance.

The different effect of active surveillance in our two screening populations reflects that the benefit of active surveillance depends on the balance between QALE gains due to averted overtreatment and QALE losses due to less curative treatment. In the average risk population, which relative to the familial risk population is characterized by slower disease progression and therefore higher risk of overdiagnosis, this balance is shifted more towards QALE gains, as the potential to reduce overtreatment is larger and the risk to miss curative treatment is smaller. In contrast, in the familial risk population with faster disease progression and lower risk of overdiagnosis, potential gains by averted overtreatment are lower and the risk to miss curative treatment is higher, which more easily shifts the benefit-harm balance of active surveillance towards QALE losses.

It was not the objective of our work to identify the optimal active surveillance strategy nor the optimal follow-up interval for active surveillance. However, additional analyses not presented in detail in this work indicate that extending the follow-up interval beyond 2 years generally worsens the benefit-harm balance of active surveillance, despite further reductions in overtreatment. A main reason why prolonged follow-up intervals yield less QALE in our model is the large drop in cure rates when moving from Gleason score below 7 (cure rate 51%) to Gleason score 7 (cure rate 30%) or higher (cure rate 11%). Based on these assumptions, each extension of the follow-up interval results in less QALE, as more men progress to Gleason score > 7 within the longer time period between two repeat biopsies, which results in harm by less curative treatment that outweighs additional benefits by additionally averted overtreatment and adverse events. To improve the potential benefits of active surveillance, criteria other than Gleason score progression to ≥7 should be chosen, which would allow treatment initiation when cure rates are still higher. Decision-analytic modeling should be used to evaluate alternative active surveillance strategies.

### Limitations

As all decision-analytic modeling studies, our decision analysis has several limitations and the results depend on valid model structure, assumptions and parameters. The main limitation of our benefit-harm analysis is that our model is strongly based on calibrated parameters, which were used to simulate the natural history of PCa in the empirically unobservable latent phase. Previously published analyses comparing a previous version of our model calibrated to prevalence, incidence and ERSPC data to a version of the previous model calibrated to incidence and ERSPC data alone have demonstrated that calibrated natural history and detection parameters vary with latent prevalence assumptions, which can strongly affect the extent of overdiagnosis, and thus the resulting benefit-harm balance of screening [41]. The latent cancer prevalence predicted with the model calibrated to latent prevalence data from autopsy studies was considerably higher than with the model calibrated without prevalence data. However, calibrated parameters for disease progression and screening sensitivity were lower, which is a compelling consequence when the observed cancer incidence is assumed to evolve from a larger prevalence pool. Both model versions differing in latent cancer prevalence were used to evaluate various screening strategies. Comparing the outputs of both models illustrated the effect of prevalence assumptions on overdiagnosis and QALE. Depending on the evaluated screening strategy, the low prevalence model predicted lifetime risks of overdiagnosis and fractions of overdiagnosis among screen-detected cancers ranging from 0.3–7% and 19–47%, respectively, whereas the high prevalence model yielded much higher estimates ranging from 1.2–16% and 77–87%, respectively. Due to the difference in overdiagnosis, the model not calibrated to prevalence data predicted QALE gains by screening, whereas the model calibrated to prevalence data predicted QALE losses. In view of our previous work, the risk of overdiagnosis in calibrated models can be understood as a function of latent prevalence, speed of disease progression, screening sensitivity, screening strategy and remaining life expectancy at time of diagnosis. Estimates of overdiagnosis predicted by our current model for men with average cancer risk considerably exceed estimates reported in the literature, which only reach up to 50% [4]. However, it should be noted that these estimates are usually derived from simulation models based on assumptions as well. For example the estimate of 50% overdiagnosis reported in the literature was derived from the 2003 MISCAN model [42], which was calibrated to the same data as our model, except for latent prevalence. Therefore, it assumed lower prevalence, faster disease progression and higher screening sensitivity, which as described before results in lower estimates of overdiagnosis and higher estimates of screening-related QALE [41]. On the other hand, the calibration of our model to latent prevalence data from autopsy studies assumes that latent cancers detected at autopsy are detectable by screening as well. This assumption may be questioned, as some autopsy-detected cancers may not affect PSA levels or may be undetectable by biopsy due to size or location. In consequence, our model might overestimate the pool of screen-detectable latent cancers, which would result in overestimation of overdiagnosis and consecutive underestimation of screening-related QALE.

Our scenario analyses suggest that screening in men with average PCa risk is not beneficial even under conservative assumptions. However, it should be noted that each scenario focused only on a single assumption. We did not perform a best case scenario analysis with all assumptions in favor of screening, which certainly would change the benefit-harm balance of screening to QALE gains.

Results for screening in men with elevated familial risk must be interpreted in view of our assumptions on the familial risk effect. Our results are based on the assumption that familial predisposition has a simultaneous effect on cancer onset and progression. This assumption might be wrong. Scenario analyses which restricted the familial risk effect to cancer onset or progression alone yielded contradictory results. This again emphasizes that assumptions about disease onset and progression, which together determine the size of the latent prevalence pool, are crucial for benefit-harm predictions from simulation models.

It should be noted that our model for men with familial predisposition assumes a twofold higher lifetime risk of PCa. In view of pooled rate ratios from a meta-analysis [51] and relative risks used in the model by Howard et al. [32] ranging from 2.5 for men with one first-degree relative with PCa to over 4 for men with more than one affected first-degree relative, our assumption may be conservative from the perspective of screening, as its net benefit was shown to increase with cancer risk. A conservative approach might be justified in areas with established PCa screening like Tyrol, because the diagnostic accuracy of family history might be reduced due to prior overdiagnosis of cancer in the family.

It also should be noted that we did not consider screening before the age of 55. As the comparison of one-time screening strategies in men with elevated familial risk indicates a trend towards less overdiagnosis and more QALE when screening is performed at age 55 rather than later, it might be possible that high risk men benefit from an even earlier screening start. Ideal starting ages in high risk men should be evaluated in further research applying more detailed risk assumptions.

Our evaluation of active surveillance focusses on a single hypothetical strategy for active surveillance. Our results clearly illustrate the interaction of important mechanisms affecting the benefit-harm balance of active surveillance in general. However, the poor performance of the evaluated strategy must not be generalized. Alternative active surveillance algorithms need to be evaluated in future modeling studies.

Concerning the credibility of our results from the familial risk model, we want to point out that elevated familial risk was modeled using a multiplicative factor on onset and progression parameters of the average risk model. This means that, in contrast to the average risk model, the high risk model was not specifically calibrated to observed stage distributions and detection rates in a high risk population. Therefore, results from that model might be surrounded with higher uncertainty than results of the average risk model.

A more general limitation of our work is that we did not study the joint uncertainty of our results using probabilistic sensitivity analysis. However, our evaluation is performed from the perspective of individual screening candidates, for whom the expected value might be considered as the only usable decision criterion, as the decision must be made, even under uncertainty. Therefore, our work focusses on deterministic sensitivity analyses investigating the influence of inter-individual variation (i.e. individual risk factors and preferences) on the optimal screening decision rather than on probabilistic analysis of joint uncertainty. Moreover, the most important drivers of our model (i.e. PCa onset and progression parameters), were derived by simultaneous calibration. Varying these highly dependent parameters in probabilistic sensitivity analysis would strongly decrease the fit of the model to observed data, which can cause arbitrary result variation unrelated to parameter uncertainty. On the other hand, a probabilistic sensitivity analysis excluding the most important model drivers would not quantify joint uncertainty appropriately, as well.

Finally, it should be noted that modeling studies can only guide patient-shared decision making. It mainly serves as a tool for communicating potential benefits and risks. The ultimate decision should be discussed with the patient considering his individual risk factors and preferences based on the best available evidence. Even though our results are unlikely to correctly reflect the absolute benefits and harms of PCa screening, given all uncertainties of the model, they reveal important trade-offs to be considered by screening candidates, physicians, decision makers and modelers.

## Conclusions

Our work shows, that the assumptions about individual PCa risk, latent PCa prevalence, and the detectability of latent cancer by screening significantly affect the benefit-harm balance of screening, and therefore screening recommendations.

The results of our modeling study suggest that PCa screening in men without strong risk factors such as familial predisposition may induce more harms than benefits. When PCa is considered in candidates with familial predisposition, individual QoL preferences and age should be carefully assessed to be able to derive individualized screening decisions to optimize the benefit-harm balance for each man.

Progression to a Gleason score of 7 may not be an ideal criterion for treatment initiation with active surveillance. Alternative criteria are needed, which permit treatment when cure rates are still high.

## Appendix

### Scenario analyses

The effect of critical parameter assumptions on benefit-harm predictions was tested in scenario analyses applying more favorable parameter assumptions for screening. We considered scenarios with no peri-operative RP mortality, shorter duration of QoL impairment due to long-term complications of treatment (i.e., reduction from 5 years to 1 year), 50% lower one-time disutility weights for biopsy and curative treatment procedures, and non-age specific utilities for men without symptomatic metastatic cancer and treatment complications (i.e., assuming a utility of one instead of age-specific utilities from the general male population). In additional scenario analyses we restricted the effect of familial risk to PCa onset and PCa progression, respectively.

Results of the scenario analyses evaluating the effect of critical model assumptions on QALDs gained versus no screening are presented in the Appendix Table 4. Analyses for men with average and elevated familial PCa risk are contrasted in the upper and lower sections of the table, respectively. To facilitate comparisons, base-case results and assumptions are also shown in the table.

Scenarios for screening in men with average PCa risk, which apply more favorable screening assumptions, still predict a negative benefit-harm balance, but with lower losses in QALE. Scenarios for screening in men with familial predisposition, applying more favorable screening assumptions, consistently yield higher gains in QALE except for one-time screening at age 69.

Assigning a utility of one to all health states without symptomatic cancer instead of age-specific utilities from the general population yields considerably higher gains in QALE in the familial risk model, but minimally affects the prediction for men with average PCa risk.

Scenario analyses investigating the effect of familial risk assumptions yield contrary results. When familial risk increases only PCa onset, the benefit-harm balance for men with familial predisposition becomes negative, whereas when only PCa progression is increased, the net benefit of screening considerably exceeds our base-case prediction.

**Table 4:** Scenario analyses – effect of model assumptions on quality-adjusted life days (QALDs) gained versus no screening

	One-time screening at age				Interval screening at age 55–59, with interval			Interval screening at age 55–64, with interval			Interval screening at age 55–69, with interval		
	55y	59y	64y	69y	4y (2 x)	2y (3 x)	1y (5 x)	4y (3 x)	2y (5 x)	1y (10 x)	4y (4 x)	2y (8 x)	1y (15 x)
Predictions for men average PCa risk													
**Base-case result**	**−1.3**	**−2.3**	**−3.9**	**−7.2**	**−2.8**	**−3.5**	**−4.4**	**−4.9**	**−5.8**	**−6.7**	**−7.3**	**−10.6**	**−11.2**
Perioperative RP mortality (base case = 0.0015)													
None	−1.2	−2.1	−3.7	−6.9	−2.5	−3.2	−4.1	−4.5	−5.3	−6.2	−6.7	−9.8	−10.3
Duration of disutility due to treatment complications (base case = 5 years)													
1 year	−0.6	−1.2	−1.9	−4.1	−1.2	−1.7	−2.3	−2.2	−2.5	−2.5	−3.0	−4.3	−3.9
One-time utility decrements (base case = age- and state-specific decrements)													
50% reduction of decrements	−0.6	−1.3	−2.2	−4.6	−1.3	−1.7	−2.2	−2.4	−2.7	−2.4	−3.4	−4.9	−4.1
Utility for non-symptomatic health states (base case = age-specific)													
Utility = 1	−1.3	−2.5	−4.1	−8.1	−2.8	−3.6	−4.6	−5.0	−5.8	−6.4	−7.3	−10.5	−10.5
Predictions for men with elevated familial PCa risk													
**Base-case result**	**1.8**	**1.7**	**0.3**	**−3.9**	**3.6**	**4.6**	**5.5**	**4.7**	**7.1**	**10.6**	**5.1**	**8.2**	**13.0**
Perioperative RP mortality (base-case = 0.0015)													
None	1.9	1.9	0.6	−3.6	3.9	4.9	5.8	5.1	7.6	11.3	5.7	9.1	14.1
Duration of disutility due to treatment complications (base case = 5 years)													
1 year	2.6	3.1	2.6	−0.6	5.4	6.7	8.0	7.8	10.9	15.8	10.0	15.6	21.9
One-time utility decrements (base case = age- and state-specific decrements)													
50% reduction of decrements	2.6	3.0	2.4	−0.9	5.3	6.7	8.0	7.6	10.7	15.7	9.6	14.9	21.3
Utility for non-symptomatic health states (base case = age-specific utility)													
Utility = 1	2.6	2.7	1.6	−3.4	5.2	6.7	8.0	7.4	10.8	16.1	8.9	14.4	21.6
Effect of familial risk factor (base case = increase of PCa onset and progression)													
Increase of PCa onset only	−2.3	−5.8	−13.1	−22.0	−5.2	−5.5	−5.3	−9.5	−8.3	−9.0	−17.0	−21.6	−19.0
Increase of PCa progression only	2.2	2.7	2.4	0.0	4.8	6.0	7.1	7.3	10.1	14.8	9.5	14.7	20.6

Results are based on individual level simulation (microsimulation) with 10 million trials. Time horizon = 120 years, Compliance = 100%. *PCa* prostate cancer, *RP* radical prostatectomy

QALDs were primary benefit-harm outcome was indicated in bold

## Abbreviations

ADT: androgen deprivation therapy
AE: Adverse event
BD: Bowel dysfunction
ED: Erectile dysfunction
ERSPC: European randomized study of screening for prostate cancer
MISCAN model: MIcrosimulation SCreening ANalysis
PCa: Prostate cancer
PCOP Model: Prostate cancer outcome and policy model
PLCO: Prostate, lung, colorectal, and ovarian cancer screening trial
PSA: Prostate-specific antigen
QALD: Quality-adjusted life day
QALE: Quality-adjusted life expectancy
QoL: Quality of life
RP: Radical prostatectomy
RT: Radiotherapy
UI: Urinary incontinence
USPSTF: United States Preventive Services Task Force
